# Fibronectin type III domain-containing protein 5 interacts with APP and decreases amyloid β production in Alzheimer’s disease

**DOI:** 10.1186/s13041-018-0401-8

**Published:** 2018-10-24

**Authors:** Yasuha Noda, Akira Kuzuya, Kyousuke Tanigawa, Mitsugu Araki, Ryoko Kawai, Biao Ma, Yoko Sasakura, Masato Maesako, Yoshitaka Tashiro, Masakazu Miyamoto, Kengo Uemura, Yasushi Okuno, Ayae Kinoshita

**Affiliations:** 1 Department of Human Health Sciences, Graduated school of Medicine, Kyoto University, 53 Kawahara-cho, Shogoin, Sakyo-ku, Kyoto, 606-8507 Japan; 2 Department of Neurology, Graduated school of Medicine, Kyoto University, 54 Shogoin kawahara-cho, Sakyo-ku, Kyoto, 606-8507 Japan; 3 Department of Pharmaceuticals, Kyoto University, 46 Shimoadachi-cho, Sakyo-ku, Kyoto, 606-8501 Japan; 4 Research and Development Group for In Silico Drug Discovery, Pro-Cluster Kobe, Foundation for Biomedical Research and Innovation (FBRI), 6-3-5, Minatojima-Minamimachi Chuo-ku, Kobe, 650-0047 Japan; 5 Neurobiology of Alzheimer’s Disease Laboratory, Massachusetts General Hospital, Harvard Medical School, 149 13th Street Charlestown, Charlestown, MA 02129 USA

## Abstract

The deposition of Amyloid-beta peptides (Aβ) is detected at an earlier stage in Alzheimer’s disease (AD) pathology. Thus, the approach toward Aβ metabolism is considered to play a critical role in the onset and progression of AD. Mounting evidence suggests that lifestyle-related diseases are closely associated with AD, and exercise is especially linked to the prevention and the delayed progression of AD. We previously showed that exercise is more effective than diet control against Aβ pathology and cognitive deficit in AD mice fed a high-fat diet; however, the underlying molecular mechanisms remain poorly understood. On the other hand, a report suggested that exercise induced expression of fibronectin type III domain-containing protein 5 (FNDC5) in the hippocampus of mice through PGC1α pathway. Thus, in the current study, we investigated a possibility that FNDC5 interacts with amyloid precursor protein (APP) and affects Aβ metabolism. As a result, for the first time ever, we found the interaction between FNDC5 and APP, and forced expression of FNDC5 significantly decreased levels of both Aβ_40_ and Aβ_42_ secreted in the media. Taken together, our results indicate that FNDC5 significantly affects β-cleavage of APP via the interaction with APP, finally regulating Aβ levels. A deeper understanding of the mechanisms by which the interaction between APP and FNDC5 may affect Aβ production in an exercise-dependent manner would provide new preventive strategies against the development of AD.

**Electronic supplementary material:**

The online version of this article (10.1186/s13041-018-0401-8) contains supplementary material, which is available to authorized users.

## Introduction

Alzheimer’s disease (AD) is pathologically characterized by senile plaques, neurofibrillary tangles, and neuronal cell death. Amyloid β (Aβ), a major component of senile plaques, is known to be cleaved from its precursor protein Amyloid precursor protein (APP) and secreted extracellularly. APP is a single transmembrane protein that is expressed in a number of different cell types, including neurons. According to the widely accepted ‘amyloid cascade hypothesis’ [1], a sequential processing of APP by β-secretase and γ-secretase leads to the generation of several types of amyloid β (Aβ). Of these, Aβ_40_ and Aβ_42_, consisting of 40 and 42 amino acids respectively, are well known as the major molecular species of Aβ. Aβ_42,_ which tends to be deposited earlier in senile plaques, is considered to be more toxic than Aβ_40._ On the other hand, sequential APP cleavages by α-secretase and γ-secretase occur in the middle of the Aβ sequence, which is considered to prevent the generation of Aβ peptides [2, 3]. Therefore, the pathway modulating Aβ production, accumulation, and degradation is critical for the pathogenesis of AD.

The largest risk factor for sporadic AD is aging, which is unavoidable; however, there are reported to be several modifiable factors including high blood pressure, diabetes mellitus, physical inactivity, low education, smoking, and so on [4]. Epidemiological and clinical studies clearly indicate that type2 diabetes mellitus elevates the morbidity rates of AD [5]. In vivo experiments using APP-overexpressing transgenic mice (AD model mice) showed that exposure to a high-fat diet or sucrose water leads to an earlier onset of cognitive deficits and pathological alterations in the brain [6]. Another group showed that voluntary exercise promotes Aβ clearance and ameliorates the activation of astrocytes and microglia in the experiments using aged mice [7]. In our previous research, we demonstrated that a high-fat diet aggravated cognitive function and amyloid pathology. Interestingly, high fat-induced Aβ deposition and memory deficit improved with a modification of lifestyle— promoting exercise and reducing the fat content of the diet. We showed that exercise is more effective in the prevention of Aβ production than dietary control through suppressing β-secretase activity and promoting the Aβ degeneration by Neprilysin [8]. Other publications have reported that the level of physical activity certainly correlates with the morbidity of AD [9]. Therefore, it is now believed that physical inactivity is one of the most attributable and modifiable risk factors of AD. As AD is considered to be type3 diabetes which only affects the brain [10], lifestyle modifications such as dietary control and exercise may regulate glucose metabolism and ameliorate the pathology of AD.

From the view point of the clearance system of Aβ through blood brain barrier, dietary control and exercise may modulate the expression of specific molecules associated with Aβ influx or efflux [11]. In AD, there is a significant reduction of low density lipoprotein receptor-related protein 1 (LRP1), a primary transporter of Aβ across the Blood-Brain Barrier (BBB) out of the brain, in the vasculature of the brain [12]. Inversely, the receptor for advanced glycation end products (RAGE) which normally transports Aβ into the brain across the BBB is demonstrated to be elevated in the microvessels in AD patients [13–15]. Intriguingly, exercise is reported to promote Aβ clearance through upregulation of LRP1 that releases Aβ into vessels from the cerebral parenchyma and through downregulation of RAGE [12, 13, 16]. These reports indicate the mechanism that lifestyle habits may directly affect Aβ pathology by modulating Aβ influx and efflux through BBB.

Aβ_42,_ a toxic form of Aβ, tends to aggregate more easily than Aβ_40_, although the exact mechanism of how it leads to neurodegeneration remains unknown so far. The hypothetical model of temporal evolution of AD proposed by Jack et al. [17] suggests that Aβ begins to accumulate in the brain long before the onset of dementia, accelerating tauopathy, that is, Aβ deposition is the most upstream event in the natural history of AD. Subsequently, another main hallmark of AD, neurofibrillary tangles, composed of hyperphosphorylated tau, begin to spread throughout the brain in the presence of senile plaques [18, 19]**.** Considering the temporal evolution of AD pathology, in order to prevent the pathological process from progression, Aβ deposition should be the primary target of therapeutics. Recent studies suggest a possibility that exercise has a protective effect on Aβ production; thus, it is now considered a promising method for modulating senile plaques. In human studies, Liang et al. reported that physically active individuals had significantly lower PIB binding, which means lower Aβ deposition in the brain [20], thus suggesting a close association between exercise engagement and brain amyloid levels.

Exercise is not only beneficial for brain function by ameliorating amyloid pathology but also by regulating glucose and lipid metabolism. Exercise may even affect adult neurogenesis in specific regions of the rodent brains, such as in the dentate gyrus of the hippocampus [21–23]. In the experiment on AD model mice, voluntary exercise induced neurogenesis which led to the improvement of learning ability [24, 25], indicating that exercise may have a beneficial effect on neurogenesis even in the presence of AD pathology.

Despite the accumulating reports on exercise intervention, the precise mechanism of its beneficial effect remains largely unknown. Recent research has shown that some peptides and proteins released from skeletal muscles during exercise affect the metabolism in other organs [26]. These secreting factors, now called “myokines”, not only act on muscles themselves in an autocrine/paracrine manner, but also mediate the interaction of muscles with other organs through endocrine mechanisms [27, 28]. Thus, skeletal muscle has recently been classified as a new endocrine organ, which secretes various kinds of myokines that are involved in the regulation of the body’s metabolic balance [28]. In respect of exercise-associated molecular pathways, one of the best-recognized molecules is the transcriptional co-activator, peroxisome proliferator-activated receptor gamma coactivator 1-alpha (PGC-1α). PGC-1α, which is localized in the heart, skeletal muscle, kidney, and to a lesser extent in the liver, pancreas, and brain, is a major regulator of exercise-induced muscle adaptation [29]. Intriguingly, PGC-1α in the brain may play an important role in synaptogenesis. Knocking down PGC-1α reduces synaptogenesis and spinogenesis in vitro and in vivo [30]. In addition, neuronal PGC1-α knockout mice present the suppression of mRNA expression of Fibronectin type III domain-containing protein 5 (FNDC5) [31]. Inversely, PGC-1α up-regulation stimulates FNDC5 expression, resulting in the deprivation of intracellular muscle ATP after exercise, which might trigger the synthesis of FNDC5.

FNDC5 is a single transmembrane protein whose mRNA is mainly expressed in skeletal muscles and in different organs, such as heart, kidney, brain, and pancreas [31]. It is proteolytically cleaved in a similar manner to PGC-1α, and secreted as the hormone “irisin”, suggesting that some of the beneficial effects of exercise could be mediated by this hormone [32]. FNDC5/irisin pathway is also upregulated in the hippocampus in a PGC-1α-dependent manner during exercise when it triggers the expression of several neuroprotective genes [32]. The release of irisin may regulate the effects of exercise on the body’s energy metabolism via an endocrine action on other tissues [33, 34], increasing energy expenditure by browning white adipocytes through mitogen-activated protein kinase p38 MAP kinase and ERK MAP kinase signaling [35] and regulating glucose metabolism through PI3K/Akt signaling pathway [36]. Animal and human studies have already shown that the levels of *Fndc5* mRNA and the circulating irisin increase after exercise, suggesting an enhancement of proteolysis of FNDC5 into irisin and the connection with fat cells where it potentially binds to a receptor to induce browning and heat production [31, 37]. There have been many studies attempting to correlate the plasma irisin levels with metabolic disorders such as obesity, diabetes, non-alcoholic fatty liver disease, and polycystic ovary syndrome; however, the results were not consistent among the various studies [38]. In studies investigating the central nervous system, there are very few reports on the FNDC5/irisin signaling pathways and their roles. It is reported that FNDC5 regulates neural differentiation like the brain derived neurotrophic factor (BDNF) [39] and that the pharmacological dose of irisin increases the proliferation of mouse hippocampal neuronal cells, which is similar to the effects of endurance exercise [40]. According to these reports, FNDC5/irisin might be a molecular mediator which plays a role in the muscle-brain crosstalk. This led us to investigate whether FNDC5 is involved in the mechanism due to which exercise has a beneficial effect on Alzheimer’s disease, in particular, on the Aβ pathology directly. This study showed for the first time a novel interaction between FNDC5 and APP, confirmed by the use of biological experiments and in silico analysis.

## Methods

### Plasmids and cell preparations

The expression vector, pcDNA3.1 was purchased from Invitrogen, and full-length APP770 tagged with V5 (APP770-V5), β-carboxyl terminal fragment tagged with myc (C99-myc) were described in our previous publication [41, 42]. Furthermore, we constructed an α-carboxyl terminal fragment tagged with myc (C83-myc) using Prime STAR mutagenesis basal kit (Takara Bio, Japan). FNDC5 protein cDNA was cloned using the muscle of wild type mice, its C-terminus additionally tagged with HA (FNDC5-HA). These were transfected into Human Embryonic Kidney 293 (HEK293) cells, and the cells and the media were retrieved after 36 h. To confirm the interaction between APP and FNDC5, we applied Aβ_1–16_ peptides (Peptide Laboratory, Japan) into the conditioned media to suppress the interaction between them. The conditioned media were exchanged to the media containing either1 μM Aβ_1–16_ peptides or Aβ_16–1_ peptides (Biologica, Japan) 8 h after transfection with APP770-V5 and FNDC5-HA in HEK293 cells. The media and the cells were retrieved 36 h after the transfection of these plasmids. These cells, suspended with 100 μl TNE buffer (10 mM Tris-HCl, 150 mM NaCl, 1 mM EDTA, 1% NP40, pH 7.8), were rotated for 1 h at 4 °C, and the soluble fraction was collected after the centrifuge at 14000 rpm for 10 min. Their media were centrifuged, and the supernatant was collected.

### Antibodies

The mouse monoclonal anti-V5-tag antibody (1:2000), mouse monoclonal anti-β-actin antibody (1:4000), mouse monoclonal anti-6E10 antibody (1:1000), rabbit polyclonal anti-APP C-terminal antibody (1:4000), and rabbit polyclonal anti-HA-tag antibody (1:1000) were purchased from Sigma (St Louis, MO). The mouse monoclonal anti-Beta amyloid (4G8) antibody (1:1000) was purchased from Bio Legend (CA, USA). The rabbit polyclonal anti-FNDC5 antibodies (1:1000) were purchased from Proteintech (IL, USA). These antibodies were used for western blotting and immunofluorescence staining. For immunoprecipitation, the mouse monoclonal anti-V5 tag antibody (MBL, Japan), the rabbit monoclonal anti-APP C-terminal (Y188) antibody (Abcam, UK), and the normal mouse or rabbit IgG were used.

### SDS-PAGE and western blotting

Targeted proteins were separated using SDS-gel (Atto, Japan) for cell lysate, followed by transference into PVDF membrane. These membranes were washed by TBS-T buffer (200 mM Tris, 1370 mM NaCl, 1% Tween, pH 7.5) three times for 5 min each and blocked by 5% skim milk diluted with TBS-T buffer for 1 h. Protein size markers were purchased from Nacalai (Japan) and Wako (Japan). Subsequently, chemiluminescence images were detected.

### Immunofluorescence staining

To confirm the location of APP770-V5 and FNDC5-HA, we examined immunofluorescence staining of SH-SY5Y cells transiently expressing these molecules. We washed prepared cells by PBS and fixed them by 4% paraformaldehyde for 15 min at room temperature. Then, these cells were permeabilized by 0.1% Triron-X and blocked by using Blocking solution (Nacalai Tesque, Japan). We used the mouse monoclonal anti-V5 antibody (1:1000; Sigma) and the rabbit polyclonal anti-HA antibody (1:1000; Sigma) for the primary antibodies to detect APP and FNDC5, and then labeled them by Alexa Fluor 594-conjugated goat anti-mouse (1:2000; Life Technologies, MA, USA) and Alexa Fluor 488-conjugated mouse anti-rabbit (1:2000; Life Technologies), respectively. As the mounting agent, we used NucBlue Fixed Cell Stain ReadyProbes reagent from Life Technologies. These cells were observed using a laser confocal scanning microscope (FV10i-LIV, Olympus, Japan).

### Computational prediction of the irisin-APP_672–699_ complex structure

The initial structural data of a transmembrane N-terminal domain of the amyloid precursor protein, APP_672–699_, was obtained from the Protein Data Bank (PDBID: 1BA4), and its conformation suitable to binding to irisin was explored using Temperature Replica-Exchange Molecular Dynamics (T-REMD) simulation [43]. Molecular Dynamics (MD) simulation of 20 ns was performed for each replica using the GROMACS 4 program [44] on a High Performance Computing Infrastructure (HPCI), and thus the total simulation time was 0.62 μs (= 20 ns × 31 replicas). A total of 300 representative APP_672–699_ structures were used for irisin- APP_672–699_ docking simulation.

The structural model of the human irisin was obtained from the Protein Data Bank (PDBID: 4LSD). After irisin- APP_672–699_ complex structures were generated using ZDOCK 3.0 program [45], we extracted 2,000 representative binding mode candidates that that satisfied the experiment-based conformational constraints. An additional file shows this in more detail (Additional file 1).

The binding stabilities of these candidates were assessed by molecular mechanics Poisson-Boltzmann surface area (MM-PBSA) [46, 47] combined with MD simulation. Each of the 2,000 irisin- APP_672–699_ docking structure models was solvated with a 150 mM NaCl aqueous solution, and its MD simulation was conducted for 10 ns under constant number of molecules, pressure, and temperature condition (298 K and 1 bar). The total simulation time was 20 μs (= 10 ns × 2,000 docking structures). After we selected the 1,620 MD trajectories in which APP_672–699_ stably bound with irisin during the 10 ns simulation, the binding free energy (*ΔG*_*bind*_) was calculated for each trajectory using the MMPBSA.py module [48] in the Amber12 package [49]. The MD-relaxed APP_672–699_ binding structure corresponding to the resulting *ΔG*_*bind*_ was calculated, and a total of 1,620 binding structures were hierarchically clustered using root-mean-square deviation of the backbone Cα atoms in the Asp672-Lys687 region, and then trees produced by the clustering were cut at a height of 10 Å. The binding stability of each conformational cluster was represented by averaging the *ΔG*_*bind*_ values corresponding to the binding structures within it. Additional details are provided in the Additional files.

### Elisa

HEK293 cells transiently expressing APP were plated at a density of 1 × 10^6^ cells/12 well dish, followed by incubation for 36 h. The aliquot of the conditioned media was collected for ELISA analysis. The peptides Aβ_40_, Aβ_42_, sAPPα, and sAPPβ in the media were measured by using Human Amyloidβ (1–40) Assay Kit, Human Amyloidβ (1–42) Assay Kit, Human sAPPα (highly sensitive) Assay Kit, and Human sAPPβ-w (highly sensitive) Assay Kit (IBL, Japan), respectively, according to the manufacturer’s instruction.

### Statistics

Signals on films were quantified with NIH Image software (National Institutes of Health). Comparison was performed using a Student’s *t*-test. For comparison of multiparametric analysis, we used one-way ANOVA, followed by the post-hoc analysis using Tukey-Kramer’s post-hoc test. Data was shown as means ± SD, and the value *p* < 0.05 was considered to indicate a significant difference. The value p < 0.05 was considered to indicate a significant difference.

## Results

### FNDC5 interacts with amyloid precursor protein

We hypothesized that exercise may modulate Alzheimer’s pathology via modulating APP metabolism. To probe for possible changes in Aβ production in response to myokines which are released from muscles, we tested whether FNDC5, glucose regulating molecules may bind to APP and affect Aβ production. Considering that endurance exercise is reported to increase the expression of FNDC5 in the hippocampus [32], we assume that increased FNDC5 may directly affect APP metabolism.

**Fig. 1:**
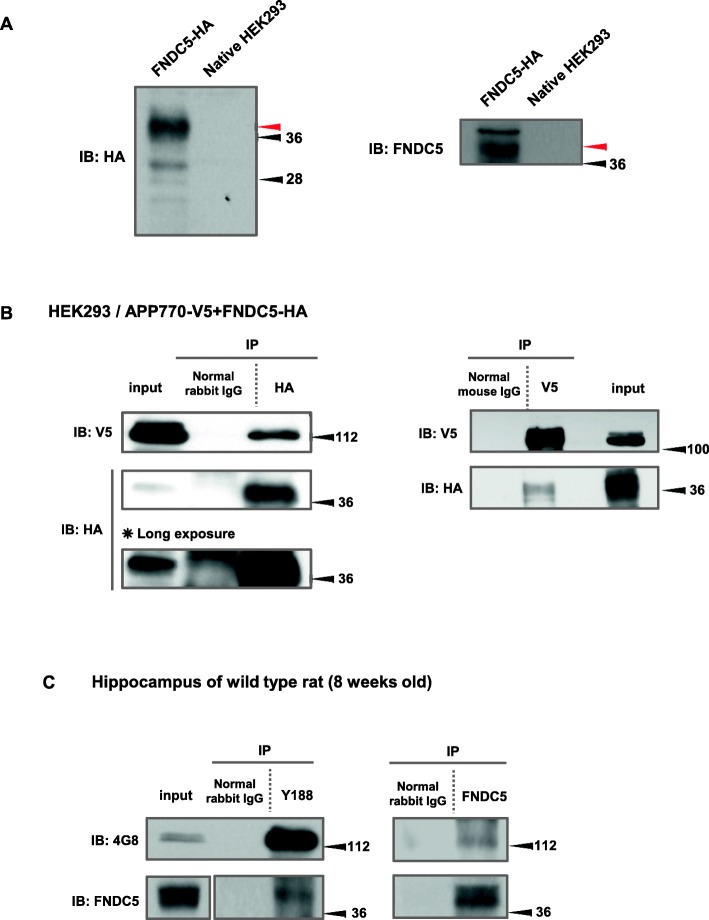
Full length of APP and FNDC5 interacted mainly at the specific domain of Aβ sequence. FNDC5 is identified by the red arrow in (**a**) and the number beside each blot is the protein standard size. In **a**, the expression of FNDC5 is identified. The left blot was blotted by anti-HA antibody and indicates the overexpressed FNDC5 tagged with HA. The right image was blotted by anti-FNDC5 antibody. The immunoprecipitation was performed in HEK293 cells transfected with APP770 tagged with V5 and FNDC5 tagged with HA. **b** shows that full length APP associates with FNDC5 in HEK293 cells. Full length APP was detected by anti-V5 antibody, and FNDC5 was detected by anti-HA antibody. **c** shows that the interaction of endogenous APP and FNDC5 was observed physiologically using the hippocampus of wild type rat (8-weeks old). The blot of 4G8 indicates APP full length

First, we verified the expression of FNDC5. The cell lysates transfected with our constructed plasmids, FNDC5-HA and native HEK293 cell, were separated (Fig. 1a). In the left image, a red arrow indicates the overexpressed FNDC5 band. In the right image, the same sample was blotted by anti-FNDC5 antibody.

**Fig. 2:**
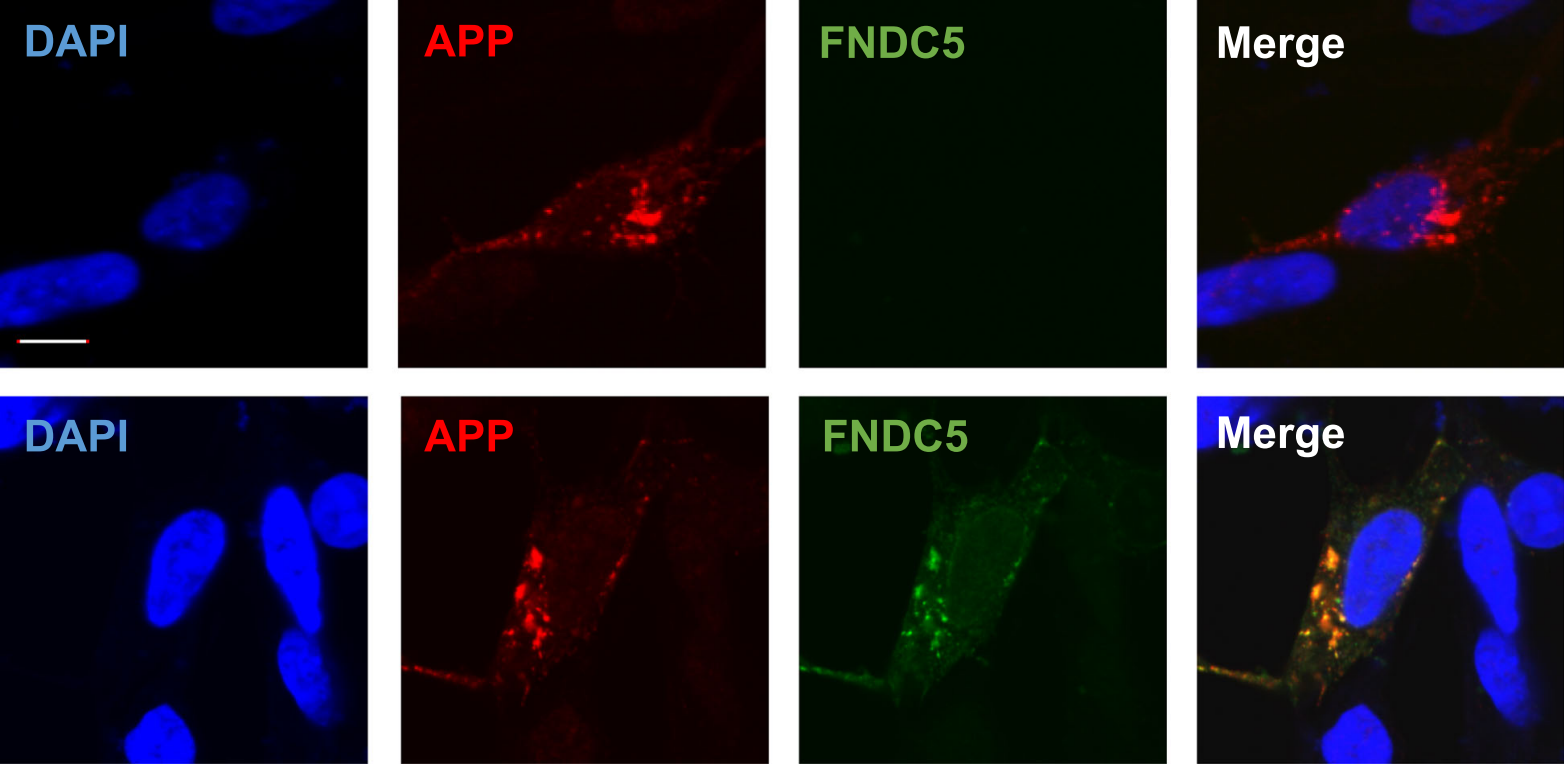
APP and FNDC5 co-localized in HEK293 cells. The images of the co-localization of APP770-V5 and FNDC5-HA were shown in Fig. 2. The panels of the upper row are the SH-SY5Y cells transfected with APP770-V5 and pcDNA3.1, and the ones of the lower row are the SH-SY5Y cells transfected with APP770-V5 and FNDC5-HA. Red and green spots indicate APP and FNDC5 localization, respectively. In the merge panel, yellow spots indicate the co-localization of APP and FNDC5. The white scale bar (in the upper panel of DAPI) indicates 10 μm

Next, we performed an immunoprecipitation assay to clarify whether FNDC5 may affect APP processing through its direct association with APP (Fig. 1b). Using HEK293 cell transfected into both of APP770-V5 and FNDC5-HA, we immunoprecipitated it by HA antibody and detected the full length of APP. The inverse approach showed comparable results. We performed these experiments five times repeatedly and obtained the same results. We also performed immunofluorescence staining and confirmed the co-localization of APP770-V5 and FNDC5-HA in SH-SY5Y cells (Fig. 2). When overexpressed, they are mostly co-localized in the cytoplasm of the cells. Using the lysates from the hippocampus of wild type rats, we detected a physiological interaction of APP and FNDC5 (Fig. 1c); these experiments were repeated three times. These results clearly revealed the interaction between APP and FNDC5 for the first time.

### FNDC5 binds amyloid precursor protein at N-terminal of Aβ sequence

**Fig. 3:**
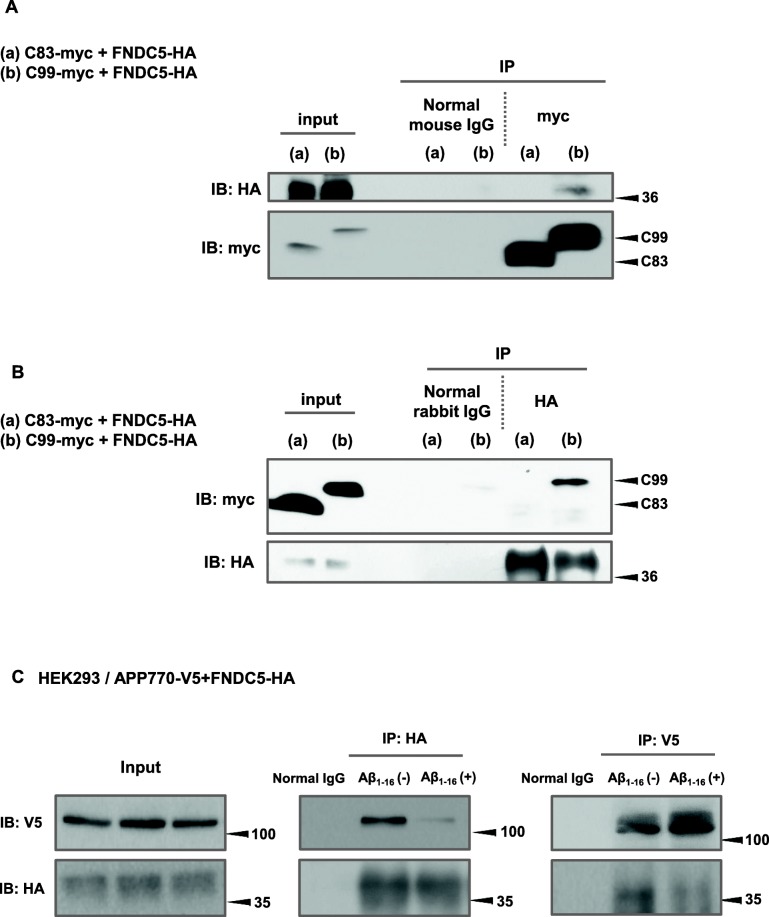
FNDC5 binds APP at the domain of Aβ sequence on N-terminus. The differential affinities of FNDC5 for C99 and C83 were indicated in (**a**, **b**). The immunoprecipitated band indicating the interaction between FNDC5 and C83 was not detected. C99 and C83 were detected by Anti-Myc antibody, and FNDC5 was detected by anti-HA antibody. **c** shows that the immunoprecipitated band between APP full length and FNDC5 is decreased with the treatment of Aβ_1–16_ peptides. For the treatment of Aβ_1–16_ (−), the reverse peptide, Aβ_16–1_ was used as a control

To specify the binding domain of FNDC5 to APP, we used APP C-terminus fragments C99 and C88 instead of full length APP. We performed immunoprecipitation and investigated which fragments of APP bind FNDC5. These results showed that FNDC5 binds C99 fragments while it does not bind to C83 (Fig. 3a, b). In order to confirm the novel interaction between APP and FNDC5, we applied 1 μM Aβ_1–16_ peptides, which competitively suppressed the binding of FNDC5 and APP. These results suggested that the primary binding domain of APP side for FNDC5 is localized between the amino acids 1–16 in N-terminal of Aβ sequence (Fig. 3c). We repeated these experiments five times respectively and obtained the same results.

### In silico simulation of the interaction between Aβ sequence and irisin

**Fig. 4:**
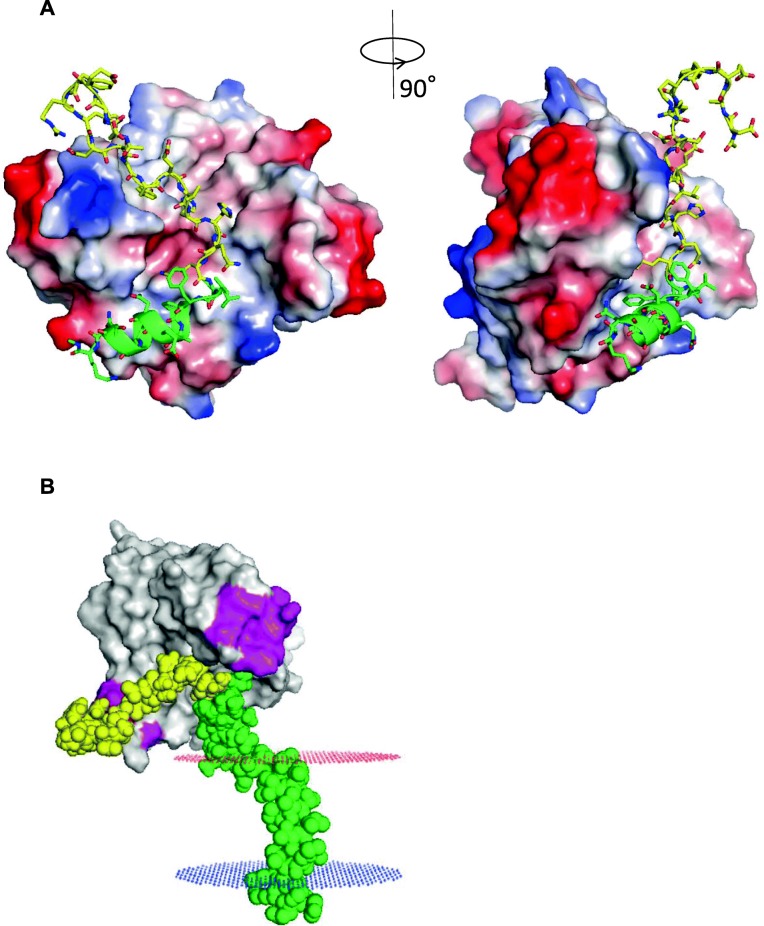
Molecular mechanism of APP recognition of irisin. **a** The APP_672–699_ binding mode on irisin predicted by computational simulations combined to experimental information. The mean of four APP_672–699_ structures assigned to the top-ranked binding mode was shown. The irisin dimer is represented by the electrostatic surface model, where electropositive, electronegative, and hydrophobic regions are colored by blue, red, and white, respectively. APP_672–699_ is represented by ribbon and stick models (green for residues 672–687 / yellow for residues 688–699, carbon; blue, nitrogen; red, oxygen). **b** Superimposition of the predicted irisin-the APP_672–699_ complex structure and that of the transmembrane domain (residues 683–728) of APP (PDBID:2LP1). Irisin and APP are represented by surface and sphere models, respectively. The putative functional loop regions in irisin (residues 30–32, 55–58, and 106–108) are colored by magenta, and residues 672–687 and 688–728 in APP are colored by yellow and green, respectively. The extracellular and intracellular membrane surfaces predicted by Orientations of Proteins in Membranes (OPM) database (ref) are depicted by red and blue dots, respectively

As a next step, we predicted a plausible binding site of the two proteins by using in silico simulation based on experimental information. Our in vitro experiments demonstrated that in Asp672-Gln687 of APP, 16 amino acids in the N-terminal sequence of C99 play a crucial role in binding with FNDC5. Also, another group previously reported that a flexible loop region in irisin, consisting of Ser30-Ser32, Glu55-Val58, and Ser106-Gln108, is associated with the recognition of other proteins [50]. We generated 2,000 irisin- APP_672–699_ binding mode candidates that satisfy this experimental information, and then extracted the 10 thermodynamically most stable ones according to the binding free energy (ΔG) based on the molecular mechanics Poisson-Boltzmann surface area (MM-PBSA) combined with the molecular dynamics simulation. An additional file shows these 10 binding modes (Additional file 2). The top-ranked binding mode with ΔG of − 26.285 ± 7.763 (kcal/mol) was shown in Fig. 4. The N-terminal sequence of C99 (Asp672-Gln687) was predicted to fit into the hydrophobic cleft between the flexible loop regions in the irisin dimer. Since no significant hydrogen bonds were observed in the predicted binding structure, bound-APP appears to be stabilized mainly by intermolecular hydrophobic interactions.

### FNDC5 expression decreased Aβ production and soluble APP β

**Fig. 5:**
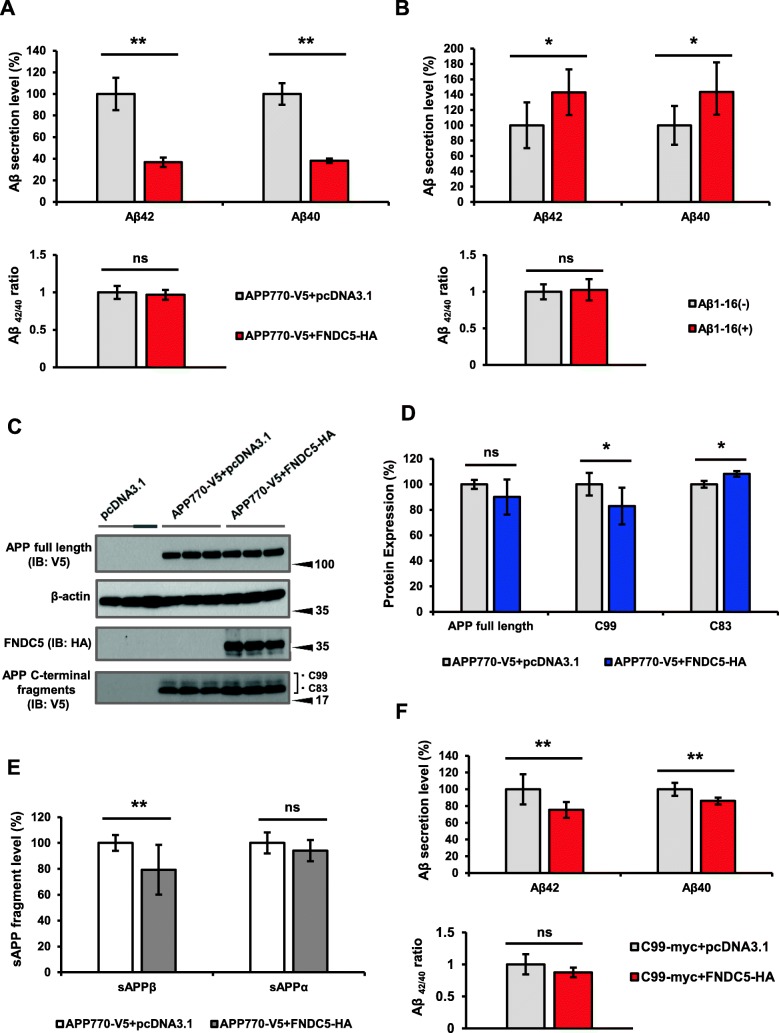
FNDC5 decreased Aβ level secreted into media in HEK293 cells. **a** indicates the results of Aβ_40_ and Aβ_42_ measurements. To compare the secretion of Aβ, the secretion level of APP770-V5 + pcDNA3.1 is referred to as 100%. In addition, we compared the ratio of Aβ_42/40_, shown in the lower panel; the ratio of APP770-V5 + pcDNA3.1 referred to as 1. The gray and red bar indicate the groups of APP770-V5 + pcDNA3.1 and APP770-V5 + FNDC5-HA, respectively. **b** shows the alteration of Aβ by the inhibition of Aβ_1–16_ peptide treatment in HEK293 cells transfected with APP770-V5 and FNDC5-HA. The secretion level of Aβ without the peptide treatment (shown as Aβ_1–16_ (−)) is referred to as 100%. The lower panel shows the ratio of secreted Aβ_42/40_ with or without the peptide treatment, where the ratio in the group of Aβ_1–16_ (−) is referred to as 1. The blots of full length APP and APP C-terminal fragments (CTF) with the expression of FNDC5 were shown in **c**, and their quantified results were shown in (**d**). The intensity of APP full length, C99 and C83 are corrected by the level of β-actin. Each band intensity in the group of APP770-V5 + pcDNA3.1 is referred to as 100%. **e** shows the alteration for sAPPβ and sAPPα in the conditioned media when APP770-V5 and FNDC5-HA was transiently transfected. The group of APP770-V5 + pcDNA3.1 is referred to as 100%. In HEK293 cells transfected with C99-myc and FNDC5-HA, a significant but less drastic decrease of Aβ in the media was shown in (**f**). The group of C99-myc + pcDNA3.1 is referred to as 100%. To compare the ratio of Aβ_42/40_, the ratio in the group of C99-myc + pcDNA3.1 is referred to as 1. As compared to the results of **a**, FNDC5 led to greater Aβ reduction in the cells with full length APP than with C99. The significances of *p*-value < 0.05 and < 0.01 were indicated as *, ** respectively

To gain further insight into the significance of the interaction between FNDC5 and APP, we explored the effect of FNDC5 on Aβ production in vitro. We transfected APP770-V5 and FNDC5-HA into HEK293 cells and measured the levels of Aβ_40_ and Aβ_42_ in the media by using ELISA. The levels of Aβ_40_ and Aβ_42_ were drastically decreased by 60%, compared to those without FNDC5 transfection (Fig. 5a). The expression of FNDC5 did not change the ratio of Aβ_42/40_. We performed these experiments four times repeatedly and the number of samples was 20. When we suppressed the interaction of these two molecules competitively by using Aβ_1–16_ peptide treatment, the amounts of Aβ_40_ and Aβ_42_ secretion were significantly reversed about 1.4 folds compared to the conditions when Aβ_1–16_ peptides were not treated (Fig. 5b). We performed these experiments three times repeatedly and the number of samples was 8. To see the effect of FNDC5 on APP expression and cleavage, we tried to detect the full length APP and APP C-terminal fragment. These detection was repeated three times the number of samples was 6. By western blotting, we confirmed that the expression of FNDC5 did not change the level of APP full length, however, tended to decrease its C-terminal fragment C99, which was compatible with its suppression effect on Aβ secretion (Fig. 5c, d). To see the effect of FNDC5 on APP metabolism, we further investigated the levels of soluble APP-β (sAPPβ) and soluble APP-α (sAPPα) by ELISA. We confirmed that the level of sAPPβ decreased significantly when FNDC5-HA was transiently expressed (Fig. 5e), whereas, it did not change the level of sAPPα. This result also supports that FNDC5 affects β-cleavage of APP, presumably by binding to the Aβ N-terminal sequence, which may contribute to the decrease of Aβ production. We performed these experiments three times repeatedly and the number of samples was 12.

To clarify the alteration of intramembranous cleavage, we tested whether the cleavage of APP-C99 fragment might be affected by FNDC5 or not. The plasmids C99-myc and FNDC5-HA were transfected into HEK293 cells, and then the production of Aβ was measured by ELISA. We performed this experiment three times repeatedly and the number of samples was 12. FNDC5 expression decreased Aβ secretion from C99 fragments significantly but less drastically than Aβ secretion from APP full length (compare Fig. 5a and f). By western blotting, we confirmed that the expression of FNDC5 did not change the level of C99 was not changed, and showed these results in an Additional file 3. This result indicates that FNDC5 may exert its effect more significantly on β-secretase than γ-secretase.

## Discussion

Dementia is the greatest global challenge for health care, especially in Japan, with the number of dementia patients being around 5.5 million. Among various diseases which cause dementia, Alzheimer’s disease is by far the most prevalent worldwide. Based on the findings of many scientific publications, it is now widely believed that lifestyle factors are closely associated with AD; thus, interventions for life style-related diseases could attenuate the progression of AD [51]. Epidemiological studies globally show that lifestyle factors, especially a physically active lifestyle, prevent or delay the onset of dementia; however, its mechanism is not clear yet. Exercise is reported to undertake neuroprotective action through the expression of the specific molecules BDNF and IGF-1, which are known to promote neurogenesis in the dentate gyrus of hippocampus in rodents. Thus, exercise might be effective in not only improving glucose homeostasis but also in maintaining the brain function in AD patients. For example, one recent report assessed the effect of a 6-week intervention of aerobic exercise program for early Alzheimer’s disease patients as a randomized controlled trial [52]. According to the report, cardiovascular fitness was correlated with changes in memory performance and bilateral hippocampal volume. This led us to ask whether peripheral processes may have beneficial effects on the brain function. More directly, how does exercise affect the pathological alteration of Alzheimer’s disease, which remains largely unknown. Therefore, we investigated whether exercise affects the early-stage pathological abnormality of Alzheimer’s disease, the deposition of Aβ. Since skeletal muscles play a pivotal role in exercise, we assume that some myokines may be involved in the process of Aβ-induced neural responses. In this paper, we focused on the exercise-associated molecule FNDC5, an irisin precursor protein, and revealed that FNDC5 may modulate Aβ production.

Recently, Xia et al. reported that FNDC5 is associated with the mechanism that Aβ oligomer suppresses the secretion of BDNF in neuro2A cells [53]. Importantly, overexpression of FNDC5 reversed the suppressive effect of Aβ on BDNF, preventing neuronal apoptosis. In vivo studies using transgenic mice supported the positive effect of intranasal-injected BDNF on cognitive decline. Furthermore, another group reported that moderate treadmill exercises could ameliorate Aβ deposition and cognitive impairment, possibly due to the PGC-1α/FNDC5/BDNF pathway [54]. Considering these results, FNDC5 probably plays the role of a critical mediator in the pathology of Alzheimer’s disease; however, the exact mechanism of how FNDC5 affects APP metabolism has not been referred yet. The present study revealed that FNDC5, which is known to be one of the myokines induced in exercise, could interact with APP, confirmed by both biochemical and in silico simulation assay for the first time. Furthermore, we speculate that FNDC5 strongly binds to the specific domain between β-secretase and α-secretase cleavage sites of APP (the amino acids 1–16 on the N-terminus of Aβ sequence). These results led us to assume that FNDC5 may affect Aβ production. Importantly, FNDC5 actually decreased the production of Aβ and sAPPβ significantly. This suggests that the expression of FNDC5 suppressed either the expression or the activity of β-secretase, prompting the proteolytic cleavage by α-secretase. The decrease of Aβ secretion from the C99 fragment by the expression of FNDC5 suggests that FNDC5 may affect γ-cleavage to some extent, but the effect on β-cleavage is significantly stronger than that on γ-cleavage. Interestingly, another group reported that the amino-terminus of C99 of APP is critical for modifying the cleavage of β-secretase through binding β-cleavage site of full length of APP and the transmembrane shedding by γ-secretase [55]. Considering these reports, our present results convincingly suggested that FNDC5 can suppress the cleavage of β-secretase by binding to the N-terminus of C99 fragment. Gathering these experiments, we presumed that FNDC5 binds the 1–16 amino acids of Aβ sequence on N-terminus. Subsequently, we examined this interaction by in silico analysis, and for the first time proposed the conformation of appropriate domain of APP and irisin domain of FNDC5. This in silico simulation datum is thought to assist our biological findings.

The exact nature of FNDC5 in the brain and the direct effect of FNDC5 on neuronal function in the nervous system have not been fully investigated. It is still unclear how FNDC5 may mediate the benefits of exercise for the brain function. In particular, it should be elucidated whether the suppressive effect on Aβ production in the present study is mediated by FNDC5 derived from peripheral tissue as a myokine or by the neuron-derived FNDC5. Previous reports indicated that FNDC5 does not cross the blood brain barrier, suggesting that FNDC5 in the brain may not be derived from the peripheral tissue. On the contrary, FNDC5 is found robustly not only in the skeletal muscles, but also in different regions of the brain [56–58]. Various publications revealed that FNDC5/irisin was detected in the Purkinje cells of the cerebellum [56], astrocytes, and microglia [59]. Especially, Wrann et al. [32] demonstrated that the increase of *Fndc5* mRNA is detected in the neurons of the cerebral cortex and the hippocampus in proportion to the amount of exercise, suggesting a possibility that exercise might increase FNDC5 expression in the brain. However, a small hormone released from FNDC5, irisin, might cross the BBB and circulate in the brain. Further in vivo study is needed to identify which one actually affects the APP metabolism in the brain.

Coinciding with our result, a muscle secretory factor, cathepsin B was recently reported to increase with running and enhance the expression of BDNF and doublecortin in adult hippocampal progenitor cells. Consistently, in cathepsin B conditional knockout mice, running exposure did not improve hippocampal neurogenesis and spatial memory function. Furthermore, exercise elevated the level of plasma cathepsin B in humans, improving hippocampus-dependent memory [60]. These results indicated that cathepsin B is an exercise-induced systemic myokine, which can enhance hippocampal neurogenesis. This report expanded our understanding of how exercise positively affects the neuronal function via peripherally released myokines.

One recent report showed that exercise did not slow cognitive impairment in people with dementia [61]. In the present study, we demonstrated that the interaction of APP and FNDC5 decreased Aβ production. Considering the cascade of AD that Aβ accumulation may start about 20–30 years before the onset of dementia, FNDC5 may play an effective role in the preclinical stage. However, when the Aβ accumulation reaches its peak and neuronal death is seen to be widespread, it may be too late to start intervention for decreasing Aβ accumulation. Taking the time course into account, we consider that the result of our study may assist the evidence for the preventive approach of AD, rather than curing it. Our results suggest that up-regulation of FNDC5 by exercise may be involved in suppressing Aβ pathology, and thus beneficial for AD prevention. Further research is required to reveal the origin of FNDC5 in the nervous system, and the exact mechanism of how exercise is beneficial for the prevention of AD via FNDC5 in vivo.

## Additional files

Additional file 1: Computational prediction of the irisin-APP_672–699_ complex structure. This is the additional technical information about in silico simulation of the interaction between irisin and APP_672–699_. (PDF 149 kb)

Additional file 2: The top ten-ranked binding modes of APP_672–699_ on irisin. The Irisin dimer and APP_672–699_ structures assigned to each conformational cluster are represented by the ribbon model. In irisin, the putative functional loop regions (residues 30–32, 55–58, and 106–108) are colored by red, and other regions are colored by orange. In APP, residues 672–687 and 688–728 are colored by cyan and blue, respectively. The binding free energy (ΔG) of each conformational cluster is calculated by averaging the values of its members and indicated as a criterion of the irisin- APP_672–699_ binding affinity. (PPTX 1729 kb)

Additional file 3: The expression of FNDC5 did not change the level of C99. These blots show that expression of FNDC5 did not change the level of C99 (A). The quantified results were shown in (B). The band intensity in the group of C99-myc + pcDNA3.1 is referred to as 100%. (PPTX 224 kb)

## Abbreviations

AD: Alzheimer’s disease
APP: Amyloid precursor protein
Aβ: amyloid –beta
BACE1: β-site APP cleaving enzyme1
BBB: Blood-brain barrier
C83: α-carboxyl terminal fragment
C99: β-carboxyl terminal fragment
FNDC5: Fibronectin type III domain-containing protein 5
