# Supplementary material for: Fibronectin type III domain-containing protein 5 interacts with APP and decreases amyloid β production in Alzheimer’s disease
Source: Mol Brain. 2018 Oct 24;11:61. doi: 10.1186/s13041-018-0401-8 (PMC6201590; doi:10.1186/s13041-018-0401-8)
Supplement: Supplementary file 1 — Computational prediction of the irisin-APP672–699 complex structure. This is the additional technical information about in silico simulation of the interaction between irisin and APP672–699. (PDF 149 kb) [file 13041_2018_401_MOESM1_ESM.pdf]

Computational system of irisin and $APP_{672-699}$

The initial structural data of the human irisin (PDBID: 4LSD) and a transmembrane N-terminal domain of the amyloid precursor protein that consists of residues Asp672-Lys699 (PDBID: 1BA4), $APP_{672-699}$, were obtained from the Protein Data Bank. Structures of flexible side chains were modeled using the structure preparation module in the Molecular Operating Environment (MOE, Chemical Computing Group, Montreal, Canada), version 2016.10. [1]. Selenomethionine in irisin was replaced with methionine. The N- and C-termini of these two protein models were capped with acetyl and N-methyl groups, respectively. The dominant protonation state at pH 7.0 was assigned for titratable residues.

Temperature Replica-Exchange Molecular Dynamics (T-REMD) simulation of $APP_{672-699}$

A nuclear magnetic resonance (NMR) study of C99 has suggested that its N-terminal region (residues 672-699) is located in the extracellular space [2]. Although this region forms a partially-helical structure in isolation, its conformation is variously-changed upon binding to other proteins [3] [4] [5] [6], suggesting that the extracellular region of C99 can be regarded as an "intrinsically disordered protein". Thus, we initially explored a $APP_{672-699}$ conformation that is suitable for binding to irisin using Temperature Replica-Exchange Molecular Dynamics (T-REMD) simulation [7] as follows. The Amber ff99SB-ILDN force-field [8] was used for irisin, $APP_{672-699}$, and ions, and TIP3P was used to model water molecules.[9] Water molecules were placed around the protein model with an encompassing distance of 8 Å, including roughly 3,000 water molecules. 150mM sodium and chloride ions were added to neutralize the system. Molecular dynamics (MD) simulations were carried out in periodic boundary conditions using the GROMACS 4 program [10] on a High Performance Computing Infrastructure (HPCI). For T-REMD simulations, a set of temperatures to obtain an exchange probability of 0.2 was generated using a Temperature generator for REMD-simulations (http://folding.bmc.uu.se/remd/) [11]. Thirty-one replicas were used with temperature ranging from 298-398K. After the fully solvated system was energy-minimized, it was equilibrated for 100 ps at individual temperatures under the constant number of molecules, volume, and temperature (NVT) condition, and run for 100ps under constant number of molecules, pressure, and temperature (NPT) condition, with positional restraints on $APP_{672-699}$ heavy atoms. The final structures were used for the subsequent T-REMD simulations under the NPT condition without the positional restraints. The replica exchange was tried every 2ps. In this procedure, the temperature of each replica was maintained with the Nose-Hoover thermostat [12, 13] and the pressure was maintained at 1 bar with the Berendsen barostat[14], where the temperature and pressure time constants were set to 0.3 ps and 1 ps, respectively. Electrostatic interactions were calculated using the particle mesh Ewald (PME) method [15] with a cut-off radius of 11Å. Van der Waals interactions were cut off at 10 Å. The P-LINCS algorithm was employed to constrain all bond lengths [16]. All

MD runs were carried out with time steps of 2 fs and snapshots were output every 2 ps to yield 500 snapshots per nanosecond of simulation. MD simulation of 20 ns was performed for each replica, and thus the total simulation time was 0.62 μs (=20 ns × 31).

We extracted 10,000 structures of $APP_{672-699}$ from T-REMD trajectory at the lowest temperature (T = 298K) every 2ps. After the backbone Cα atoms were structurally aligned, tertiary structures of these atoms in the 10,000 snapshots were clustered into 300 categories by using the k-means clustering method. For each clustering category, the structure that has the smallest root-mean-square deviation from the cluster center was selected as a representative one. A total of 300 representative $APP_{672-699}$ structures were further used for following irisin- $APP_{672-699}$ docking simulation.

Irisin-$APP_{672-699}$ docking simulation and binding free energy estimation

Our *in vitro* experiments clearly demonstrated that an N-terminal region of C99 (Asp672-Gln687) is required for binding to irisin. Also, a crystallographic analysis of irisin suggested that its flexible loop regions (Ser30-Ser32, Glu55-Val58, and Ser106-Gln108) play a significant role in recognition of other proteins [17]. Based on these experimental information, we predicted a plausible binding structure of $APP_{672-699}$ to irisin as follows.

ZDOCK 3.0 program [18] was used to generate candidates of the irisin- $APP_{672-699}$ complex structure. With the standard default settings, each of the 300 $APP_{672-699}$ structures was docked into irisin and the 100 first-ranked binding poses were output, generating a total of 30,000 complex structure models. Assuming that the N-terminal region of C99 (Asp672-Gln687) and the flexible loops in irisin are involved in their binding, we selected 15,009 $APP_{672-699}$ binding-mode candidates that satisfy following two conditions: (i) the flexible loop regions in irisin are located within 5Å of $APP_{672-699,}$ and (ii) more than 15% of APP atoms consisting of residues Asp672-Gln687 interacted closely with irisin (<5Å). Conformational clustering of these $APP_{672-699}$ docking-poses was then performed on its backbone Cα atom coordinates to categorize them into 2,000 representative binding-modes using the k-means clustering method. In each clustering category, the docking pose that has the smallest root-mean-square deviation from the cluster center was selected as a representative one. The binding stabilities of these irisin- $APP_{672-699}$ complex structure models were assessed by molecular mechanics Poisson-Boltzmann surface area (MM-PBSA) [19] [20] combined with MD simulation.

MD simulation of irisin in complex with $APP_{672-699}$ was performed as follows. Each of the 2,000 irisin-$APP_{672-699}$ complex structure models was set to the initial structure. Force fields for protein, water, and ion molecules were the same as described above. Water molecules were placed around the complex model with an encompassing distance of 8 Å, including roughly 19,000 water molecules. 150mM sodium and chloride ions were added to neutralize the system. After each of the

fully solvated systems was energy-minimized, it was equilibrated for 100 ps under the NVT condition, and run for 100 ps under the NPT condition, with positional restraints on irisin and $APP_{672-699}$ heavy atoms. Production runs were conducted under the NPT condition (298K and 1bar) without the positional restraints, using simulation parameters described in the previous section. A 10ns production run was performed for each of the 2,000 irisin-$APP_{672-699}$ docking structure models. The total simulation time was 20 μs (= 10ns ×2,000 docking structures). Among the 2,000 MD trajectories, we selected 1,620 in which $APP_{672-699}$ stably binds to irisin during the 10ns simulation by judging whether the complex structure after 10ns satisfies the above-described two conditions.

The MM-PBSA calculation was carried out using the MMPBSA.py module [21] in the Amber12 package [22]. In this method, the protein-ligand binding free energy (ΔG_{bind}) is calculated according to the following equation:

$$\Delta G_{bind} = \Delta E_{gas} - T\Delta S + \Delta G_{solv}$$

where ΔE_{gas} is the molecular mechanics energy difference in the gaseous phase, T is absolute temperature, ΔS is the conformational entropy, and ΔG_{solv} is the solvation free energy. ΔE_{gas} and ΔG_{solv} were calculated by the single trajectory approach [23], in which the (free) energies for “complex”, “protein”, and “ligand” are computed from an MD trajectory for the protein-ligand (irisin- $APP_{672-699}$) complex only. In contrast, ΔS was computed using three individual MD trajectories of the complex, protein, and ligand, respectively, because small-molecular size compounds and peptides exhibit larger differences in conformational flexibility between the solvated and protein-bound states [23]. The MM-PBSA calculation was performed using a set of 450 structures extracted from a trajectory from 1 to 10 ns at regular intervals. $T\Delta S$ was calculated by the quasi-harmonic approximation using the same trajectory. Ionic strength for a series of calculations was set to 150 mM. This protocol was performed for each of the 1,620 trajectories. Since several initial docking poses of $APP_{672-699}$ significantly changed during the 10 ns simulation, the mean binding-structure corresponding to the resulting ΔG was calculated by averaging the structures observed from 1 to 10 (ns), named “MD-mean binding structure”. After the backbone Cα atoms in irisin were structurally aligned, a total of 1,620 MD-mean binding structures of $APP_{672-699}$ were hierarchically clustered using root-mean-square deviation of the backbone Cα atoms in the Asp672-Lys687 region, and then trees produced by the clustering were cut at a height of 10Å. The binding stability of each conformational cluster was calculated by averaging the ΔG_{bind} values corresponding to the MD-mean binding structures within it.

<References>

1. Goto J, Kataoka R, Muta H, Hirayama N: **ASEDock-docking based on alpha spheres and excluded volumes.** *Journal of chemical information and modeling* 2008, **48:**583-590.

2. Barrett PJ, Song Y, Van Horn WD, Hustedt EJ, Schafer JM, Hadziselimovic A, Beel AJ, Sanders CR: **The amyloid precursor protein has a flexible transmembrane domain and binds cholesterol.** *Science* 2012, **336:**1168-1171.

3. Shen Y, Joachimiak A, Rosner MR, Tang WJ: **Structures of human insulin-degrading enzyme reveal a new substrate recognition mechanism.** *Nature* 2006, **443:**870-874.

4. Crespi GA, Hermans SJ, Parker MW, Miles LA: **Molecular basis for mid-region amyloid-beta capture by leading Alzheimer's disease immunotherapies.** *Sci Rep* 2015, **5:**9649.

5. Rauth S, Hinz D, Borger M, Uhrig M, Mayhaus M, Riemenschneider M, Skerra A: **High-affinity Anticalins with aggregation-blocking activity directed against the Alzheimer beta-amyloid peptide.** *Biochem J* 2016, **473:**1563-1578.

6. Hoyer W, Gronwall C, Jonsson A, Stahl S, Hard T: **Stabilization of a beta-hairpin in monomeric Alzheimer's amyloid-beta peptide inhibits amyloid formation.** *Proc Natl Acad Sci U S A* 2008, **105:**5099-5104.

7. Sugita Y, Okamoto Y: **Replica-exchange molecular dynamics method for protein folding.** *Chem Phys Lett* 1999, **314:**141-151.

8. Lindorff-Larsen K, Piana S, Palmo K, Maragakis P, Klepeis JL, Dror RO, Shaw DE: **Improved side-chain torsion potentials for the Amber ff99SB protein force field.** *Proteins* 2010, **78:**1950-1958.

9. Jorgensen WL, Chandrasekhar J, Madura JD, Impey RW, Klein ML: **Comparison of simple potential functions for simulating liquid water.** *J Chem Phys* 1983, **79:**926-935.

10. Hess B, Kutzner C, van der Spoel D, Lindahl E: **GROMACS 4: Algorithms for Highly Efficient, Load-Balanced, and Scalable Molecular Simulation.** *Journal of Chemical Theory and Computation* 2008, **4:**435-447.

11. Patriksson A, van der Spoel D: **A temperature predictor for parallel tempering simulations.** *Phys Chem Chem Phys* 2008, **10:**2073-2077.

12. Nose S: **A molecular dynamics method for simulations in the canonical ensemble.** *Mol Phys* 1984, **52:**255-268.

13. Hoover WG: **Canonical dynamics: Equilibrium phase-space distributions.** *Phys Rev A: At Mol Opt Phys* 1985, **31:**1695-1697.

14. Berendsen HJC, Postma JPM, van Gunsteren WF, DiNola A, Haak JR: **Molecular dynamics with coupling to an external bath.** *J Chem Phys* 1984, **81:**3684-3690.

15. Darden T, York D, Pedersen L: **Particle mesh Ewald: An N·log(N) method for Ewald sums in large systems.** *J Chem Phys* 1993, **98:**10089-10092.

16. Hess B, Bekker H, Berendsen HJC, Fraaije JGEM: **LINCS: A linear constraint solver for molecular simulations.** *J Comput Chem* 1997, **18:**1463-1472.

17. Schumacher MA, Chinnam N, Ohashi T, Shah RS, Erickson HP: **The structure of irisin reveals a novel intersubunit beta-sheet fibronectin type III (FNIII) dimer: implications for receptor activation.** *J Biol Chem* 2013, **288:**33738-33744.

18. Pierce BG, Hourai Y, Weng Z: **Accelerating protein docking in ZDOCK using an advanced 3D convolution library.** *PLoS One* 2011, **6:**e24657.

19. Massova I, Kollman PA: **Computational alanine scanning to probe protein-protein interactions: A novel approach to evaluate binding free energies.** *J Am Chem Soc* 1999, **121:**8133-8143.

20. Kollman PA, Massova I, Reyes C, Kuhn B, Huo SH, Chong L, Lee M, Lee T, Duan Y, Wang W, et al: **Calculating structures and free energies of complex molecules: Combining molecular mechanics and continuum models.** *Acc Chem Res* 2000, **33:**889-897.

21. Miller BR, 3rd, McGee TD, Jr., Swails JM, Homeyer N, Gohlke H, Roitberg AE: **MMPBSA.py: An Efficient Program for End-State Free Energy Calculations.** *Journal of chemical theory and computation* 2012, **8:**3314-3321.

22. Case DA, Darden TA, Cheatham ITE, Simmerling CL, Wang J, Duke RE, Luo R, Walker RC, Zhang W, Merz KM, et al: *AMBER 12.* University of California, San Francisco; 2012.

23. Hou T, Wang J, Li Y, Wang W: **Assessing the performance of the MM/PBSA and MM/GBSA methods. 1. The accuracy of binding free energy calculations based on molecular dynamics simulations.** *Journal of chemical information and modeling* 2011, **51:**69-82.
