# Supplementary material for: Fibronectin type III domain-containing protein 5 interacts with APP and decreases amyloid β production in Alzheimer’s disease
Source: Mol Brain. 2018 Oct 24;11:61. doi: 10.1186/s13041-018-0401-8 (PMC6201590; doi:10.1186/s13041-018-0401-8)
Supplement: Supplementary file 2 — The top ten-ranked binding modes of APP672–699 on irisin. The Irisin dimer and APP672–699 structures assigned to each conformational cluster are represented by the ribbon model. In irisin, the putative functional loop regions (residues 30–32, 55–58, and 106–108) are colored by red, and other regions are colored by orange. In APP, residues 672–687 and 688–728 are colored by cyan and blue, respectively. The binding free energy (ΔG) of each conformational cluster is calculated by averaging the values of its members and indicated as a criterion of the irisin- APP672–699 binding affinity. (PPTX 1729 kb) [file 13041_2018_401_MOESM2_ESM.pptx]

## Slide 1
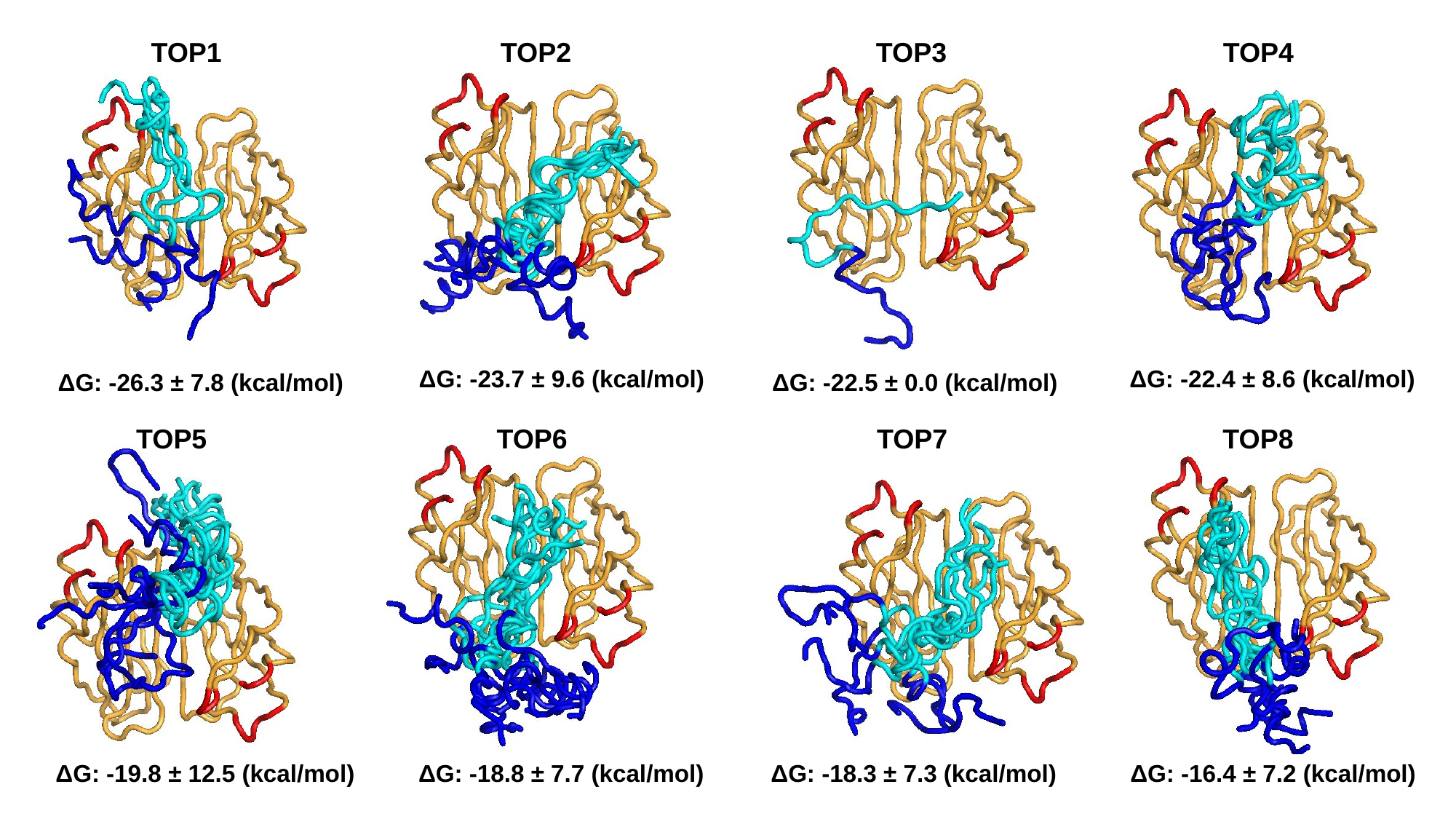

TOP1
TOP2
TOP3
TOP4
ΔG: -23.7 ± 9.6 (kcal/mol)
ΔG: -22.4 ± 8.6 (kcal/mol)
ΔG: -26.3 ± 7.8 (kcal/mol)
ΔG: -22.5 ± 0.0 (kcal/mol)
TOP8
TOP5
TOP6
TOP7
ΔG: -19.8 ± 12.5 (kcal/mol)
ΔG: -18.8 ± 7.7 (kcal/mol)
ΔG: -18.3 ± 7.3 (kcal/mol)
ΔG: -16.4 ± 7.2 (kcal/mol)

## Slide 2
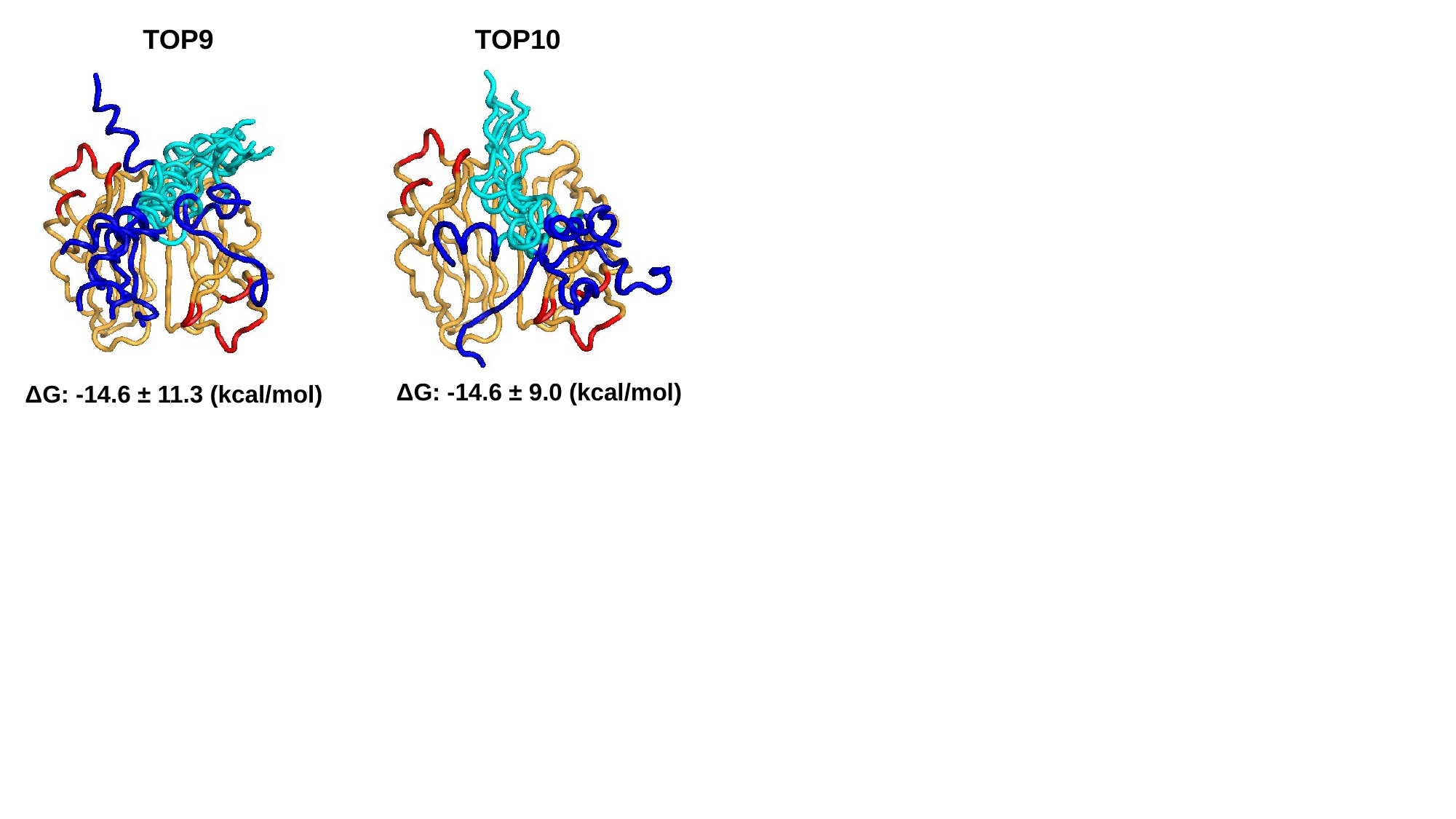

TOP9
TOP10
ΔG: -14.6 ± 9.0 (kcal/mol)
ΔG: -14.6 ± 11.3 (kcal/mol)
