# Supplementary material for: Fibronectin type III domain-containing protein 5 interacts with APP and decreases amyloid β production in Alzheimer’s disease
Source: Mol Brain. 2018 Oct 24;11:61. doi: 10.1186/s13041-018-0401-8 (PMC6201590; doi:10.1186/s13041-018-0401-8)
Supplement: Supplementary file 3 — The expression of FNDC5 did not change the level of C99. These blots show that expression of FNDC5 did not change the level of C99 (A). The quantified results were shown in (B). The band intensity in the group of C99-myc + pcDNA3.1 is referred to as 100%. (PPTX 224 kb) [file 13041_2018_401_MOESM3_ESM.pptx]

## Slide 1
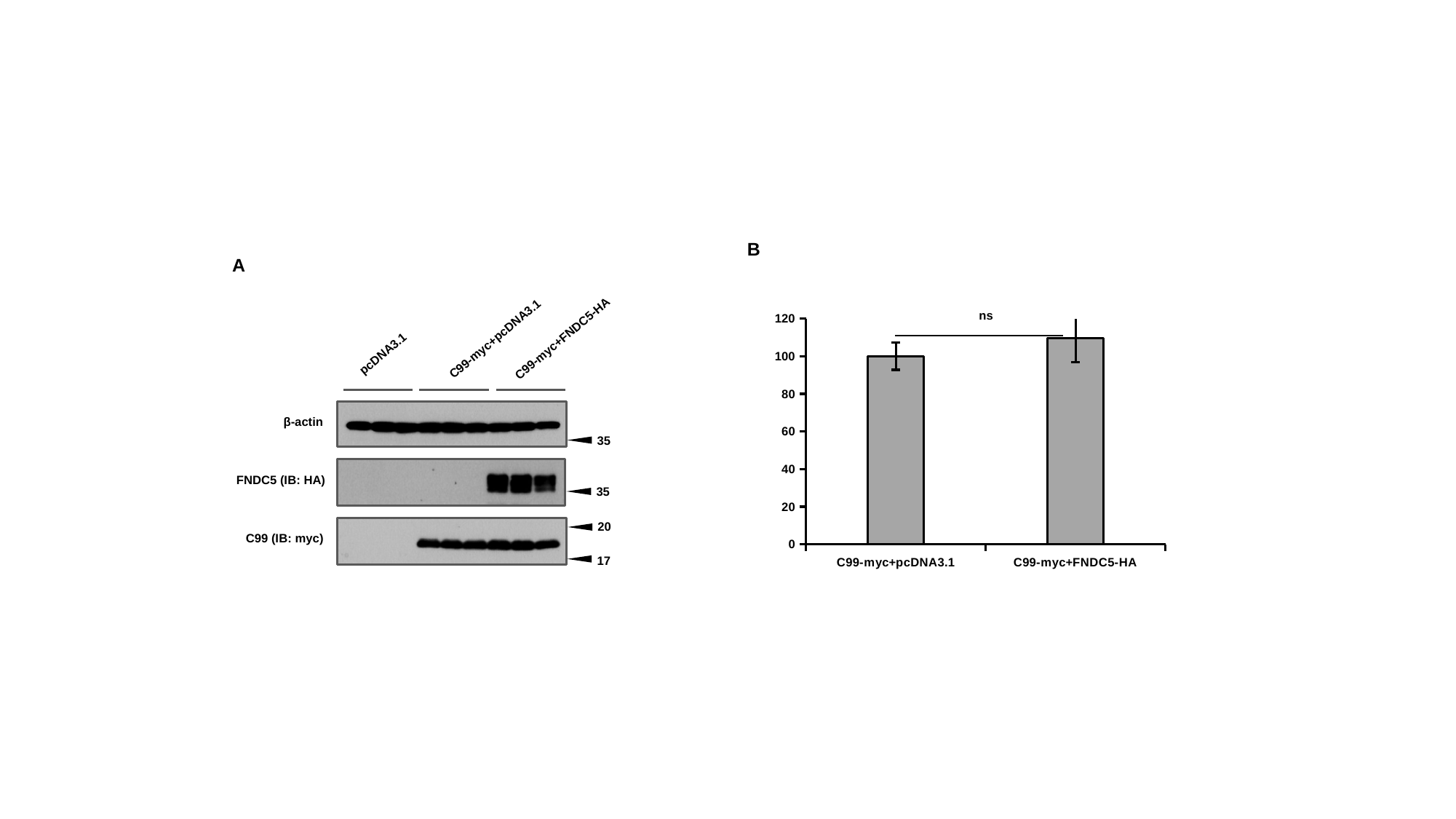

B
ns
### Chart
| Category | |
|---|---|
| C99-myc+pcDNA3.1 | 100.0 |
| C99-myc+FNDC5-HA | 109.625975545247 |A
C99-myc+FNDC5-HA
C99-myc+pcDNA3.1
pcDNA3.1
β-actin
35
FNDC5 (IB: HA)
35
20
C99 (IB: myc)
17
